# 670-nm light treatment reduces complement propagation following retinal degeneration

**DOI:** 10.1186/1742-2094-9-257

**Published:** 2012-11-26

**Authors:** Matt Rutar, Riccardo Natoli, Rizalyn Albarracin, Krisztina Valter, Jan Provis

**Affiliations:** 1 The John Curtin School of Medical Research, College of Medicine, Biology and Environment, The Australian National University, Building 131, Garran Rd, Canberra, ACT 2601, Australia; 2 ARC Centre of Excellence in Vision Science, The Australian National University, Canberra, ACT 2601, Australia; 3 ANU Medical School, The Australian National University, Canberra, ACT 2601, Australia

## Abstract

**Aim:**

Complement activation is associated with the pathogenesis of age-related macular degeneration (AMD). We aimed to investigate whether 670-nm light treatment reduces the propagation of complement in a light-induced model of atrophic AMD.

**Methods:**

Sprague–Dawley (SD) rats were pretreated with 9 J/cm^2^ 670-nm light for 3 minutes daily over 5 days; other animals were sham treated. Animals were exposed to white light (1,000 lux) for 24 h, after which animals were kept in dim light (5 lux) for 7 days. Expression of complement genes was assessed by quantitative polymerase chain reaction (qPCR), and immunohistochemistry. Counts were made of C3-expressing monocytes/microglia using *in situ* hybridization. Photoreceptor death was also assessed using outer nuclear layer (ONL) thickness measurements, and oxidative stress using immunohistochemistry for 4-hydroxynonenal (4-HNE).

**Results:**

Following light damage, retinas pretreated with 670-nm light had reduced immunoreactivity for the oxidative damage maker 4-HNE in the ONL and outer segments, compared to controls. In conjunction, there was significant reduction in retinal expression of complement genes C1s, C2, C3, C4b, C3aR1, and C5r1 following 670 nm treatment. *In situ* hybridization, coupled with immunoreactivity for the marker ionized calcium binding adaptor molecule 1 (IBA1), revealed that C3 is expressed by infiltrating microglia/monocytes in subretinal space following light damage, which were significantly reduced in number after 670 nm treatment. Additionally, immunohistochemistry for C3 revealed a decrease in C3 deposition in the ONL following 670 nm treatment.

**Conclusions:**

Our data indicate that 670-nm light pretreatment reduces lipid peroxidation and complement propagation in the degenerating retina. These findings have relevance to the cellular events of complement activation underling the pathogenesis of AMD, and highlight the potential of 670-nm light as a non-invasive anti-inflammatory therapy.

## Introduction

Age-related macular degeneration (AMD) is a global epidemic with an estimated worldwide prevalence of 30 to 50 million [1], and a leading cause of blindness in people aged over 65 (reviewed in [2]). While anti-vascular endothelial growth factor (VEGF) therapy has proved to be of benefit for neovascular or ‘wet’ AMD [3], there are presently no treatments that mitigate progression of photoreceptor loss in the more common atrophic or ‘dry’ form of AMD [4]. Although the pathogenesis of AMD is a complex multifactorial process, the recent discovery of a direct association of complement activation with the incidence of AMD has firmly established inflammation as an important factor mediating its pathogenesis [5,6].

The complement system is a component of the innate immune response, which provides a rapid host defense against a range of immunological challenges, and aiding in the maintenance of homeostasis [7,8]. Complement activation initiates a cascade of proteolytic cleavages [9,10], which augment the ability of the host to initiate humoral defenses against infectious pathogens [11], and promote the removal of potentially noxious substances including extracellular debris [7,9,10,12], immune complexes [8,13-15], and apoptotic cells [14,16-19]. When activated and poorly regulated, however, complement may induce the destruction of host tissue (reviewed in [20,21]). In AMD, a pathogenic role of the complement system has been revealed through a number of gene association studies. These identified a significant association between the Y402H sequence variant in the regulatory gene complement factor H (CFH) with the incidence of AMD [22-25], as well as other susceptibility variants in complement pathway genes C2 [5,26], CFB [5,26], and the central component C3 [27-31]. While the precise cellular events that promote complement activity in the degenerating retina are unclear (reviewed in [5]) it is known that oxidative damage to photoreceptors promotes the activation of complement and deposition of C3 protein, as documented in a carboxyethylpyrrole (CEP)-mediated mouse model of AMD [32,33].

Previous investigations have indicated that oxidative damage may be modulated by exposure to irradiation with 670-nm red light. Exposure to light concentrated in the red to near-infrared light range (630 to 1,000 nm) is known to react with the redox active metal centers of cytochrome c oxidase (a key constituent of the electron transport chain) that results in increased electron transfer and improved mitochondrial respiration, and ATP synthesis [34-36]. Studies have indicated that 670-nm light exposure reduces oxidative damage in models of rotenone-induced neurotoxicity [37], optic nerve transection [38], and 2,3,7,8-tetrachlorodibenzo-p-dioxin (TCDD)-induced toxicity [39]. Moreover, 670-nm light irradiation has been shown to accelerate wound healing [40], and ameliorate tissue damage in models of Parkinson’s disease [41], multiple sclerosis [42], and methanol-induced retinal toxicity [43].

We have previously shown that retinal irradiation with 670-nm light attenuates photoreceptor apoptosis induced by exposure to bright continuous white light (BCL) in rats [44], a model with pathogenic features in common with the atrophic ‘dry’ form of AMD [45-49]. Additionally, we have shown that a suite of complement-related genes are upregulated following BCL exposure, and that C3 is expressed in the retina by infiltrating monocytes/microglia [50]. However, the effect of 670-nm light irradiation on complement propagation in the degenerating retina has not been explored. In the current study we aimed to investigate the expression and localization of complement genes, in correlation with oxidative damage, following 670-nm light treatment and light damage. Our data show that pretreatment with 670-nm light reduces the expression of complement components and receptors including in the retina following BCL exposure. Further, we find a reduction in the recruitment of C3-expressing microglia/macrophages in the retina following 670-nm light, in spatiotemporal coincidence with decreases in the oxidative damage marker 4-hydroxynonenal (4-HNE) [51].

## Methods

### Animals and light exposure

All experiments conducted were in accordance with the Association for Research in Vision and Ophthalmology (ARVO) Statement for the Use of Animals in Ophthalmic and Vision Research. Adult Sprague–Dawley (SD) rats were born and reared in dim cyclic light conditions (12 h light, 12 h dark) with an ambient light level of approximately 5 lux.

Prior to BCL exposure, some animals were preconditioned with 670-nm light using a WARP75 670 nm LED array (QBMI Photomedicine, Barneveld, WI, USA) while others were sham treated. Animals in the treatment group were exposed to 9 J/cm^2^ of 670-nm light for 3 minutes daily over a period of 5 days, according to our protocol described previously [44]. All animals were then dark adapted for a minimum of 15 h then transferred to individual cages designed to allow light to enter unimpeded. BCL exposure commenced consistently at 9:00 am, and was achieved using a cold-white fluorescent light source positioned above the cages (18 W, Cool White; TFC, Taipei, Taiwan), at an intensity of approximately 1,000 lux at the cage floor. BCL exposure was maintained for 24 h, after which animals were immediately returned to dim cyclic conditions for a post-exposure period of 7 days. This timepoint was chosen for analysis since maximal upregulation of C3, recruitment of C3-expressing monocytes/microglia, and formation of the lesion occurs at this time [50]. Age-matched dim-reared animals, either treated or sham treated with 670 nm, served as controls. Animals were then killed and retinal tissue obtained for analysis.

### Tissue collection and processing

Animals were killed with an overdose of barbiturate administered by an intraperitoneal injection (60 mg/kg bodyweight, Valabarb; Virbac, Milperra, Australia). The eye from some animals was marked at the superior surface for orientation then enucleated and processed for cryosectioning, while the retina from others was excised through an incision in the cornea and prepared for RNA extraction.

Eyes for cryosectioning were immediately immersion fixed in 4% paraformaldehyde in 0.1 M phosphate-buffered saline (PBS) (pH 7.3) for 3 h at room temperature, then processed as previously described [46], and cryosectioned at 16 μm. Retinas for RNA extraction were immediately immersed in chilled RNAlater solution (cat. no. 7024; Ambion, Austin, TX, USA), then stored in according to the manufacturer’s instructions. RNA was then extracted from each sample and analyzed following previously established methodology [52,53].

### Quantitative real-time polymerase chain reaction (qPCR)

First-strand cDNA synthesis was performed using a protocol described previously [53]. Gene amplification was measured using commercially available TaqMan® hydrolysis probes (Applied Biosystems, Foster City, CA, USA), the details of which are provided in Table 1. The hydrolysis probes were applied following a previously established qPCR protocol [53]. The fold change was determined using the ΔΔC_q_ method. Expression of the target gene was normalized relative to the expression of the reference gene glyceraldehyde-3-phosphate dehydrogenase (GAPDH), which has shown no appreciable change following BCL exposure previously [50].

**Table 1:** TaqMan probes used

**Gene symbol**	**Gene name**	**Catalog**	**Entrez gene ID**
C1s	Complement component 1, s subcomponent	Rn00594278_m1	NM_138900.1
C2	Complement component 2	Rn00597176_m1	NM_172222.2
C3	Complement component 3	Mm00437858_m1	NM_009778.2
C3ar1	Complement component 3a receptor 1	Rn00583199_m1	NM_032060.1
C4-1 (C4b)	Complement component 4, gene 1 (C4B)	Rn00709527_m1	NM_031504.2
C5r1	Complement component 5a receptor 1	Rn02134203_s1	NM_053619.1
GAPDH	Glyceraldehyde-3-phosphate dehydrogenase	Rn99999916_s1	NM_017008.3

### *In situ* hybridization

To investigate the localization of C3 mRNA transcripts in the retina following BCL, a riboprobe to C3 was generated for *in situ* hybridization on cyrosections of retinal tissue, as described in previous studies conducted by our group [50,54]. The C3 riboprobe was hybridized overnight at 57°C, and then washed in saline sodium citrate (pH 7.4) at 60°C.

### Outer nuclear layer (ONL) thickness measurements

Thickness of the ONL in each age group was measured in increments of 1 mm along the full length of retinal cyrosections cut in the parasaggital plane (superioinferior), which were in close proximity to the vertical meridian. The DNA-specific dye bisbenzamide (Calbiochem, La Jolla, CA, USA) was used to visualize the cellular layers. ONL thickness was calculated as the ratio of ONL thickness to the distance between the outer and inner limiting membranes (OLM-ILM), to take into account any obliquely cut sections. The total ONL ratio from each retina is the average of two retina sections at comparable locations.

### Immunohistochemistry

Cryosections from each group were used for immunohistochemistry with an antibody against complement C3 (1:50, cat. no. ab11887; Abcam, Cambridge, MA, USA), 4-HNE (1:200, cat. no. HNE11-S; Alpha Diagnostic, San Antonio, TX, USA), and ionized calcium binding adaptor molecule 1 (IBA1) (1:1,000, cat. no. 019–19741; Wako, Osaka, Japan). Immunohistochemistry was performed using methodology previously described [53]. Immunofluorescence was viewed using a Zeiss laser scanning microscope (Carl Zeiss, Jena, Germany), and acquired using PASCAL software (Zeiss, v4.0). Images were prepared for publication using Adobe Photoshop software.

### Quantification of C3-expressing nuclei

Counts of C3-expressing nuclei were performed on retinal cryosections stained for C3 using *in situ* hybridization (as described above); identification of these C3-expressing nuclei as monocytes/microglia was confirmed in a previous investigation by our group [50]. Counts of C3-expressing nuclei were carried out along the full length of retinal sections cut in the parasaggital plane (superoinferior) close to the vertical meridian, in adjacent fields measuring 1 mm across.

### Statistical analysis

Statistical analysis was performed using one-way analysis of variance (ANOVA) with Tukey’s multiple comparison post test. For each analysis, differences with a *P* value <0.05 were considered statistically significant.

## Results

### Quantification of ONL thickness following BCL exposure and 670-nm light treatment

**Figure 1:**
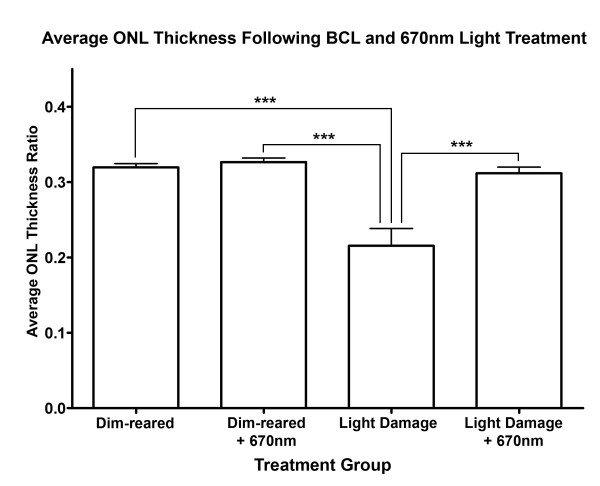
**Measurements of outer nuclear layer (ONL) thickness following 670-nm light treatment and bright continuous white light (BCL) exposure.** The thickness of the ONL decreased significantly following exposure to BCL, compared to both dim-reared and dim-reared + 670 nm groups (*P* <0.05). In comparison, the ONL was significantly thicker in animals treated with 670-nm light prior to BCL exposure (*P* <0.05). Dim reared n = 3, dim reared + 670 nm n = 3, light damage n = 3, light damage + 670-nm light n = 3; error bars represent SEM. *Significant change using analysis of variance (ANOVA) with Tukey’s post test where *P* <0.05.

The effect of 670-nm light pretreatment on photoreceptor survival following BCL was assessed using ONL thickness measurements across the retina (Figure 1). The average thickness ratio of the ONL decreased by 27% following exposure to BCL, compared to dim-reared animals (*P* <0.05). In contrast, animals treated with 670-nm light prior to BCL exposure showed substantial preservation of ONL thickness compared to those exposed to BCL alone (*P* <0.05), which was comparable to ONL thickness of dim-reared animals (*P* >0.05). No change was observed in ONL thickness in dim-reared animals treated with 670-nm light, compared to untreated controls (*P* >0.05).

### Immunoreactivity for 4-HNE in the retina

**Figure 2:**
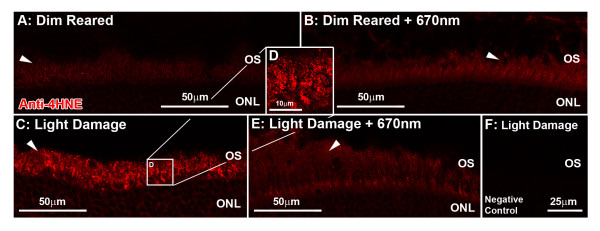
**Immunoreactivity (IR) for 4-hydroxynonenal (4-HNE; red) in the retina following 670-nm light treatment and exposure to bright continuous white light (BCL).** (**A**,**B**) IR for 4-HNE was faintly detected in the inner (IS) and outer segments (OS) of both untreated (**A**) and 670-nm light-treated (**B**) dim-reared animals (arrows). (**C**,**D**) IR for 4-HNE was more intense in the IS and OS (arrow) following exposure to BCL. (**E**) Treatment with 670-nm light prior to BCL resulted in a marked reduction in IR for 4-HNE (arrow), compared to light damage alone (**C**). (**F**) Negative controls showed no specific staining for 4-HNE following BCL exposure. ONL = outer nuclear layer; OS = outer segments.

Immunoreactivity (IR) for 4-HNE was detected in the inner (IS) and outer (OS) segments at low levels in both 670-nm light and untreated control retinas (Figure 2A,B, arrow). Following exposure to BCL, IR for 4-HNE was more intense (Figure 2C,D) and formed multiple deposits throughout the IS and OS region area (arrow). In animals treated with 670-nm light prior to BCL however, levels of 4-HNE IR were lower the IS and OS region area (Figure 2E), and more comparable to dim reared.

### Modulation of complement-related gene expression with 670-nm light pretreatment

**Figure 3:**
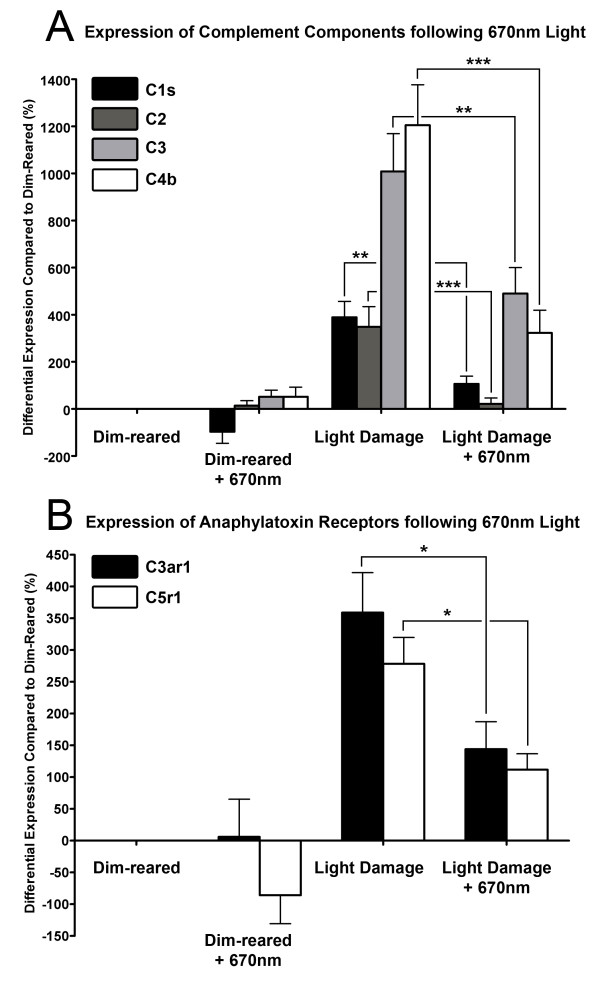
**Expression of complement-related genes in the retina by quantitative polymerase chain reaction (qPCR) following 670-nm light treatment and bright continuous white light (BCL) exposure.** (**A**,**B**) The expression of complement components C1s, C2, C3, C4b (**A**) and receptors C3aR1, C5r1 (**B**) increased significantly following BCL relative to dim-reared animals (*P* <0.05). In animals treated with 670-nm light prior to BCL exposure, the expression of all complement-related genes assessed was substantially reduced, compared to those exposed to BCL alone (*P* <0.05). Dim-reared animals exposed 670-nm light showed no significant modulation of complement gene expression relative to controls (*P* >0.05). Dim reared n = 3, dim reared + 670 nm n = 3, light damage n = 3, light damage + 670-nm light n = 3; error bars represent SEM. *Significant change using analysis of variance (ANOVA) with Tukey’s post test where *P* <0.05.

We examined the expression of six complement-related genes using qPCR (Figure 3) that were identified following BCL in microarray analysis conducted by us previously [50]; these include complement components C1s, C2, C3, and C4b, as well as anaphylatoxin receptors C3aR1, and C5r1. Following exposure to BCL, the expression of components C1s, C2, C3, C4b increased significantly compared to dim reared (*P* <0.05, Figure 3A). Differential expression of C3, and C4b peaked at 1,009% and 1,204% following BCL, while C1s and C2 showed more modest increases (389% and 348% respectively). C3aR1 and C5r1 also showed robust increases in expression following BCL exposure (359% and 278% respectively, *P* <0.05, Figure 3B). In animals treated with 670-nm light prior to BCL, the expression of all complement components (Figure 3A) and receptors (Figure 3B) assessed was substantially lower than those subjected to BCL alone (*P* <0.05). The differential expression of C2 in particular was reduced to only 21% in the 670-nm light-treated BCL group, and was statistically indistinguishable from dim-reared animals (*P* >0.05). There was no significant change in the expression of complement genes in dim-reared animals treated with 670-nm light, compared to untreated controls (*P* <0.05).

### Localization of C3 mRNA and protein in the retina following BCL and 670-nm light treatment

**Figure 4:**
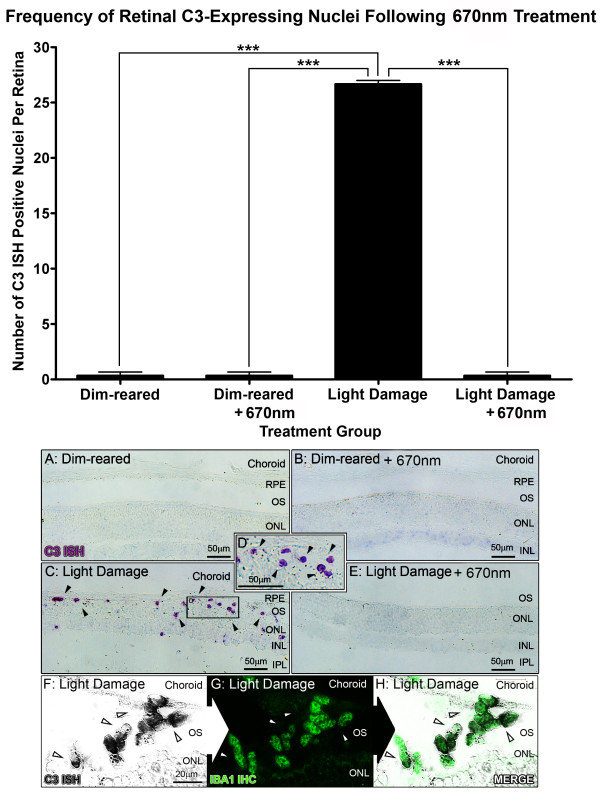
***In situ *hybridization for C3 mRNA in the retina following 670-nm light and bright continuous white light (BCL) exposure.** (**A**-**E**) Representative images from the superior mid-periphery show *in situ* hybridization for C3 mRNA in the retina. Retinas from dim-reared (**A**) and 670-nm light-treated dim-reared (**B**) animals showed no staining for C3 mRNA in the retina except for infrequent C3-expressing nuclei associated with the retina vasculature (data not shown). In BCL-exposed animals (**C-D**) C3-expressing nuclei were more numerous in the outer nuclear layer (ONL) and outer segments within the lesion area, while none were observed in those treated with 670-nm light prior to BCL exposure (**E**). (**F**,**G**) C3 expression (dark grey) in sections counterimmunolabelled with anti-IBA1 (green), showing immunoreactivity in C3-expressing nuclei within the degenerating ONL (arrows) following BCL. Histogram: Quantification of C3-expressing nuclei per retina showed a dramatic increase from near zero in dim-reared animals to 26.7 following BCL exposure (*P* <0.05). In contrast, the number of C3-expressing nuclei was reduced to near zero in animals pretreated with 670-nm light following BCL (*P* <0.05). Dim reared n = 3, dim reared + 670-nm light n = 3, light damage n = 3, light damage + 670-nm light n = 3; error bars represent SEM. *Significant change using analysis of variance (ANOVA) with Tukey’s post test where *P* <0.05.

Because of its central role in the activation and propagation of complement, we selected C3 for further investigation in relation to 670-nm light treatment. The localization of C3 expression in the retina was assessed with *in situ* hybridization (Figure 4). C3 was expressed in the retina by C3-expressing nuclei IR for the monocyte/microglia marker IBA1 (Figure 4C-F), consistent with our previous investigation [50]. C3-expressing nuclei were sparsely distributed in the retinal vasculature in dim-reared animals, at near zero per retina on average (Figure 4A, histogram). Following exposure to BCL, we detected a substantial increase (*P* <0.05) in the number of C3-positive cells to 26.7 per retina (Figure 4, histogram), which were mostly associated with the degenerative remains of photoreceptors in the ONL (Figure 4C,D, arrows). In contrast, pretreatment with 670-nm light resulted in a dramatic reduction in the number of C3-expressing nuclei to near zero following BCL (*P* <0.05), consistent with dim-reared animals.

**Figure 5:**
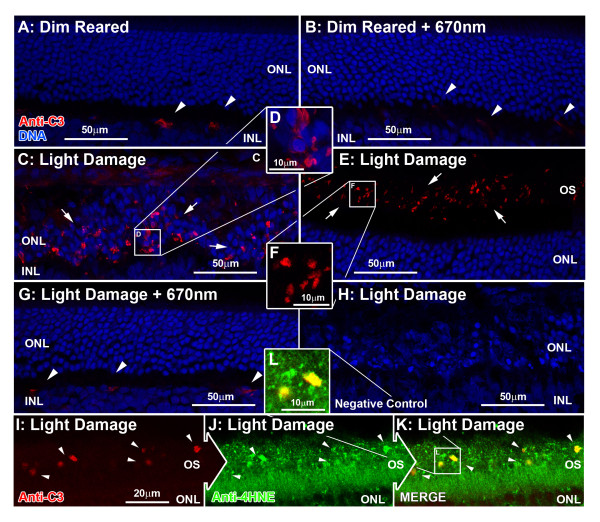
**Immunoreactivity (IR) for C3 (red) in the retina following 670-nm light treatment and exposure to bright continuous white light (BCL).** (**A**-**F**) Representative images of C3 immunoreactivity taken from the superior mid-periphery. (**A,B**) IR for C3 was faintly detected in retinal vasculature (arrows) of dim-reared retinas (**A**), as well as those treated with 670-nm light (**B**), at comparable levels of IR. (**C-F**) Following BCL exposure, IR for C3 was observed in multiple deposits throughout the outer nuclear layer (ONL) in the lesion area (**C-D**, arrows) and the layer of outer segments at the edge of the lesion (**E-F**, arrows). (**G**) C3-IR was vastly reduced in the ONL and outer segments of animals treated with 670-nm light prior to BCL, and appeared similar to dim-reared controls (arrows). (**H**) Negative controls showed no specific staining for C3 following BCL exposure. (**I**-**L**) C3-IR (red) in the outer segments of sections counterimmunolabelled with anti-4-hydroxynonenal (4-HNE) (green), shows colocalization for aggregations of 4-HNE immunoreactivity (arrows) following BCL. C = choroid; ONL = outer nuclear layer; OS = outer segment layer.

IR for C3 protein was performed using an antibody against C3 (Figure 5A-J). C3 was detected faintly within the retinal vasculature (Figure 5A, arrows), and the choroid (data not shown) of dim-reared animals. Following BCL exposure we detected strong C3-IR in multiple deposits throughout the ONL (Figure 5C,D, arrows) and the layer of outer segments (Figure 5E,F, arrows) at the site of the developing lesion. Using coimmunolabelling for 4-HNE, we observed colocalization between C3-IR deposits in the outer segment layer and aggregations of 4-HNE IR (Figure 5I-L, arrows) in animals exposed to BCL alone. In animals treated with 670-nm light prior to BCL however, C3-IR was nearly absent from in the ONL and outer segments (Figure 5G), with a distribution similar to dim-reared controls (Figure 5G, arrows).

## Discussion

The results of this study demonstrate the efficacy of irradiation with 670-nm light in suppressing lipid peroxidation and complement propagation following BCL exposure through several novel findings. First, we show that 670-nm light irradiation reduces immunoreactivity for 4-HNE in the inner and outer segment region following BCL exposure. Second, using qPCR we show that 670-nm light pretreatment inhibits the expression of a suite of complement genes following BCL, including components from the classical pathway (C1s, C2, C3, C4b), and anaphylatoxin receptors (C3aR1, C5r1). Third, we show a decrease in C3-expressing monocytes/microglia to the ONL following 670-nm light pretreatment, which coincides with reduced deposition of C3 protein in the ONL and photoreceptor outer segments.

Previous investigations, including our own, have shown that irradiation of the retina with various 670-nm light paradigms reduces photoreceptor degeneration following exposure to BCL [44,55]. The current study, however, is the first to show that retinal pretreatment with 670-nm light reduces the expression of complement-related genes following BCL exposure. These include classical components C1s, C2, and C4b, which mediate assembly of the C3 convertase [9], as well as macrophage receptor genes C3aR1 and C5r1 that recognize cleavage products of C3 and C5 respectively [20]. Furthermore, we show that irradiation with 670-nm light reduces the recruitment of microglial cells that synthesize and deposit C3 protein in the outer retina following BCL exposure. Our findings are also consistent with a previous study demonstrating a reduction IBA1 positive microglia in aging mice following irradiation with 670-nm light [36]. While complement activation has beneficial properties such as aiding debris clearance by recruited phagocytes (reviewed in [10]), other experimental evidence suggests that robust complement propagation may be detrimental following injury. Indeed, a study using mice deficient in the regulatory gene complement factor D (CFD) indicates that complement propagation exacerbates photoreceptor death following light damage [56].

The present findings suggest that 670-nm light pretreatment attenuates oxidative damage to photoreceptors and reduces inflammation, which may in turn reduce stimulation of the complement cascade. These effects of 670-nm light may be due to an interaction with mitochondria, since compromised mitochondrial function may initiate proinflammatory signaling pathways, and generation of reactive oxygen species (reviewed in [57]). This is supported by the findings of Kokkinpoulos and colleagues, who found that 670 nm irradiation increases mitochondrial membrane polarization (and consequently and ATP synthesis) *in vitro*, and reduces deposition of C3 in aged mice [36].

Irradiation with 670-nm light has previously been shown to reduce markers of oxidative damage, such as manganese superoxide dismutase (MnSOD), in other degenerative models [37-39], and our current findings indicate that 670-nm light therapy decreases production of 4-HNE, a byproduct of lipid peroxidation [58], in photoreceptors following BCL exposure. We also find deposition of C3 occurs in spatiotemporal coincidence with increases in 4-HNE following BCL exposure. This is consistent with the investigations by Hollyfield and colleagues, in which mice immunized with the oxidative damage byproduct CEP develop AMD-like retinal degeneration and show increased deposition of complement C3 in the outer retina [32,33]. Several investigations also show that exposing RPE cultures to photo-oxidative stress, or to oxidized photoreceptor outer segments, reduces their ability to express the complement regulatory gene CFH [59,60], thus promoting complement activation.

The mechanism by 670 nm modulates the recruitment of C3-expressing microglia is unclear, though it may be related to reduction of oxidative stress and/or reduced expression of proinflammatory factors. Irradiation with 670-nm light reduces expression of the proinflammatory cytokine tumor necrosis factor α (TNFα) in the retina of aged mice [36], and the chemokine Ccl2 (a chemoattractant for monocytes/microglia [61,62]) following light damage [63]. Furthermore, accumulation of lipofuscin constituents, such as the bisretinoid A2E, may promote activation and deposition of complement by microglia *in vitro*, by simultaneously reducing synthesis of the inhibitor CFH while increasing synthesis of CFB, a promoter of the alternative pathway [64]. Increased generation of C3 activation products has also been observed in RPE cells that have accumulated photo-oxidative products of A2E [65].

### Relevance to complement propagation in human retinal degeneration

Our findings, in conjunction with the results from other investigations, have previously shown that light damage in rats shares pathological features in common with ‘dry’ AMD [45-49]. Like the widely used laser-induced model of neovascular AMD, this model employs an acute damaging stimulus to evoke long-term, site-specific changes in the retina. Although the rat retina lacks a macula and its key specialization the fovea centralis, it includes a feature that is homologous to the foveal region: the area centralis, in the superiotemporal portion of the retina [66-68]. Following light damage this region develops focal degeneration of photoreceptors and RPE cells, and associated changes to the blood-retinal barrier, which mimics many histopathological aspects of advanced ‘dry’ AMD [45-48].

Complement activation is well established in the literature as a key factor in the pathogenesis of AMD. Key among these are gene association studies which show an association of polymorphisms in a range of complement-related genes with the pathogenesis of AMD (reviewed in [5]), including a string of investigations which have identified a strong association with C2 [5,26] and C3 [27-31]. Additionally, histological analyses of post-mortem AMD eyes indicate that complement components and regulatory proteins are present in drusen deposits (reviewed in [5]), particularly activation products of C3 such as C3d and C3b [69-71]. We show in our investigation that irradiation of the retina with 670-nm light substantially reduces the expression C2 and C3, as well as the deposition of C3 protein in the outer retina, following light-induced degeneration. Moreover, mitochondrial dysfunction and oxidative damage are thought to be involved in the pathogenesis of AMD [72-74], and several recent investigations have shown levels of oxidative biomarkers are elevated in patients with AMD, compared to controls [75,76]. As such, 670-nm light irradiation may have potential as a non-invasive intervention to reduce activation of complement in neural degenerations including atrophic AMD.

## Conclusions

Our findings suggest that retinal irradiation with 670-nm light substantially reduces the propagation of complement in the retina following light-induced degeneration, and is associated with a concomitant reduction in oxidative damage to the photoreceptors. Consequently, these findings further clarify the role of complement and oxidative damage in retinal degeneration, and suggest that 670-nm light irradiation may be a useful strategy to control detrimental propagation of inflammatory responses in retinal degenerations including atrophic AMD.

## Competing interests

The authors declare that they have no competing interests.

## Authors’ contributions

MVR designed and conducted the experiments, conducted the analysis, and wrote the paper; RCN designed the experiments; RA conducted the experiments; KV designed the experiments; and JMP designed the experiments, and wrote the paper. All authors read and approved the final manuscript.
